# The Effects of Sperm and Seminal Fluid of Immunized Male Mice on In Vitro Fertilization and Surrogate Mother–Embryo Interaction

**DOI:** 10.3390/ijms221910650

**Published:** 2021-09-30

**Authors:** Galina Vladimirovna Kontsevaya, Ludmila Alekseevna Gerlinskaya, Yury Mikhailovich Moshkin, Margarita Vladimirovna Anisimova, Aliya Konstantinovna Stanova, Tatyana Ivanovna Babochkina, Mikhail Pavlovich Moshkin

**Affiliations:** 1 Federal Research Center Institute of Cytology and Genetics, Siberian Branch of RAS, 630090 Novosibirsk, Russia; koncevayagalina@bionet.nsc.ru (G.V.K.); yury.moshkin@gmail.com (Y.M.M.); rita.medvedeva89@gmail.com (M.V.A.); aliya.stanova@mail.ru (A.K.S.); mmp@bionet.nsc.ru (M.P.M.); 2 Biological Institute at Tomsk State University, 634050 Tomsk, Russia

**Keywords:** reproduction, in vitro fertilization, immune stimulation, sperm, seminal fluid

## Abstract

The latest vaccination campaign has actualized the potential impact of antigenic stimuli on reproductive functions. To address this, we mimicked vaccination’s effects by administering keyhole limpet hemocyanin (KLH ) to CD1 male mice and used their sperm for in vitro fertilization (IVF). Two-cell embryos after IVF with spermatozoa from control (C) or KLH-treated (Im) male mice were transferred to surrogate mothers mated with vasectomized control (C) or KLH-treated (Im) male mice, resulting in four experimental groups: C–C, Im–C, C–Im, and Im–Im. The pre-implantation losses were significantly lower in the Im–C group than in the C–Im group. At the same time, the resorption rates reduced markedly in the C–Im compared to the Im–C group. Embryo and placenta weights were significantly higher in the Im–Im group. Although the GM-CSF levels were lower in the amniotic fluid of the gestating surrogate mothers in the Im–Im group, they were strongly correlated with embryo mass. The number–size trade-off was only significant in the Im–Im group. This suggests a positive, cooperative effect of spermatozoa and seminal fluid from immune-primed males on embryo growth and the optimal distribution of surrogate mother maternal resources despite the negative impact of males’ antigenic challenge on the IVF success rate.

## 1. Introduction

Adverse environmental factors affect the reproductive success of males and their offspring’s physiological and behavioral characteristics [1,2,3,4]. Among these are strange antigens from pathogenic and non-pathogenic microorganisms, food, etc., to which the mammalian habitat is exposed. Infections negatively affect sperm quality (concentration, motility, etc.) due to (1) the generation of reactive oxygen species (ROS) in the seminal fluid [5] and (2) a rise in pro-inflammatory cytokines in the male genital tract [6]. Since the immune response to any antigen includes the activation of innate and adaptive immunity, it could be assumed that any pathogenic and non-pathogenic antigens that cause inflammation and trigger antibody production may affect male fertility and offspring health. Thus, large-scale vaccination campaigns during pandemics might act as a prominent population-wide immunological factor impacting the reproductive system in humans. At the same time, little is yet known about the transgenerational consequences of the antigen stimulation addressed to paternal adaptive immunity.

Recent studies have shown that activation of male mice’s systemic immunity induced by a single administration of protein keyhole limpet hemocyanin (KLH) before mating affects embryo growth and development [5,6,7]. The increase in embryo weight and the fetal–placental index observed upon mating with antigen-stimulated male mice positively correlated with an increase in the embryotrophic granulocyte-macrophage colony-stimulating factor (GM-CSF) in the amniotic fluid of pregnant female mice [7]. In addition, the effects of KLH administration were conferred to adult offspring. At the ages of 12–14 weeks, the offspring of immunized male mice showed: (1) a predominance of excitatory neurotransmitters in the amygdala of the brain; (2) an increase in the mass index of immunocompetent organs (thymus, spleen); (3) an increase in the levels of blood androgens after antigen stimulation [8].

Changes in spermatogenesis due to the fact of local inflammation in the testes are most often considered the cause of fathers’ immune priming impact on offspring. Testis cells express pathogen-associated molecular pattern (PAMP) receptors such as Toll-like receptors (TLRs). A single administration of TLR agonists, such as lipopolysaccharide (LPS), peptidoglycan (PG), or polyinosinic:polycytidylic acid (polyIC), to C57BL/6 males before mating induced local inflammation in the testes. This also led to epigenetic changes in embryonic development and offspring during the feeding period [9]. LPS triggers the local production of pro-inflammatory cytokines in the testes, such as TNF-α, IL-1β, and IL-6, affecting sperm motility, concentration, and male fertility [9,10,11].

Besides spermatozoa, a paternal immunization signal could be conferred to offspring through seminal fluid. On the one hand, the seminal fluid directly modulates the parameters of the sperm [12]. On the other hand, it presents male antigens to the female through an interaction with the epithelium of the female reproductive tract [13,14]. In particular, it has been shown that individual variations in the concentration of anti-KLH IgG in seminal fluid correlate with placental mass [8]. Therefore, it is hypothesized that the paternal reproductive and transgenerational effects of immunization are determined by a combination of influences of immune signals on the parameters of sperm and seminal fluid.

In this work, we further explored this idea by studying the impact of cross-talk between adaptive immunity, sperm, and seminal fluid on in vitro fertilization (IVF) success and embryo growth. The study was performed on sexually mature (i.e., 12–14 weeks old) CD1 outbred mice to account for genetic diversity, which modulates individual characteristics of the immune response. We show that the immune priming of male mice by KLH lowered the spermatozoa number and IVF success rate. IVF with the spermatozoa of antigen-stimulated males decreased cell division and survival of in vitro cultivated blastocysts. Therefore, the immune stimulation of males reduced the fertilization capacity of their spermatozoa. To further dissect the cross-talk between males’ adaptive immunity and reproduction, we obtained two-cell embryos by IVF with spermatozoa from control (C) and immunized (Im) male mice, which were transferred to surrogate mothers mated with vasectomized control (C) or KLH-treated (Im) males. Although the independent effects of sperm and seminal fluid of immunized males on embryo growth were not statistically significant, their combined effect resulted in a marked increase in the mass of embryos on day 16 of pregnancy. Thus, the adverse effects of male immunization on spermatozoa were overruled by immune signals conferred to the seminal fluid and, together, they stimulated embryo growth. This suggests a significant impact of paternal immune activation on fertilization capacity and offspring development relayed through both the spermatozoa and seminal fluid.

## 2. Results

### 2.1. Impact of the Male Mice Antigenic Challenge on Spermatozoa

**Figure 1:**
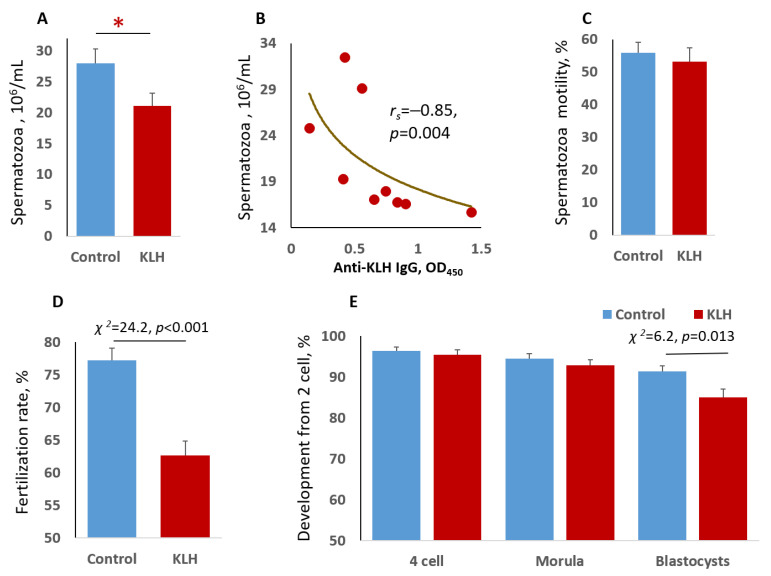
Effects of immunization on the number, motility, and in vitro fertilization rate of spermatozoa from control and KLH CD1-treated male mice. (**A**) Sperm concentration differed significantly between groups. * Student’s *t*-test, *p* < 0.05. (**B**) Correlation of sperm concentration and serum anti-KLH IgG in KLH-treated males. (**C**) Percentage of motile sperm. (**D**) Fertilization rate expressed as a percentage of two-cell embryos out of the total number of oocytes (496 in control and 490 in the KLH-treated groups). (**E**) Percent of embryos developed in vitro from two cells (*n* = 383 in control and *n* = 307 in the KLH-treated groups) to blastocysts. Between-group differences were evaluated by χ^2^-test in (**D**,**E**).

On day 7, after the immunization of CD1 male mice (12–14 weeks old) with KLH, we detected anti-KLH IgG serum at a concentration of 0.68 ± 0.12 OD450 (*n* = 9), which corresponds to the previous study [8]. Morphometric (i.e., size and elongation) and kinematic (i.e., velocities and frequencies) parameters of spermatozoa collected seven days after injection with KLH or saline (control) did not differ significantly between groups (Table 1). Although pro-inflammatory cytokines were expected to affect sperm quality, their concentrations only increased in the first few days of the immune response and leveled off after 5–8 days [15,16]. At the same time, the concentration of spermatozoa was lower after the immune priming of male mice with KLH (Figure 1A), suggesting suppression of male germline maturation by cytokines [17]. This correlated negatively with individual variability in the immune response (concentration of the serum anti-KLH IgG) to antigenic challenge (Figure 1B). Spermatozoa motility did not change significantly due to the immune stimulation (Figure 1C). Thus, male antigenic challenge had only a modest impact on spermatogenesis.

### 2.2. The Impact of the Male Mice Antigenic Challenge on IVF and Blastocyst Cell Divisions

**Figure 2:**
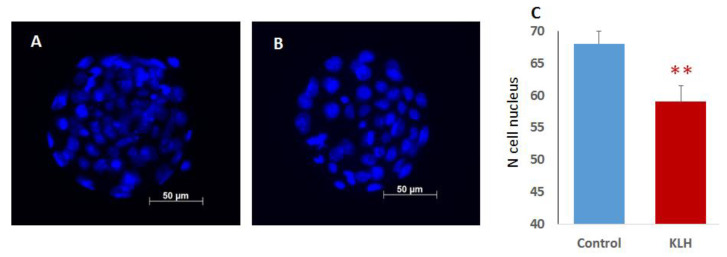
The number of cells (nuclei) in blastocysts of the control and KLH groups. (**A**,**B**) Representative blastocysts of the control (**A**) and KLH (**B**) groups stained by Hoechst-33342. (**C**) The number of nuclei that differed significantly between groups. ** *p* < 0.01, Student’s *t*-test.

To address the fertilization potential of spermatozoa from the KLH-treated males, we performed IVF experiments with oocytes collected from virgin CD1 females (12–14 weeks old). The females were superovulated with mare’s serum gonadotropin and human chorionic gonadotropin administered sequentially (see Section 4, Materials and Methods). The KLH-treated males showed a lower fertilization rate than the control as was judged from the ratio of two-cell embryos to the total number of oocytes (Figure 1D). The number of two-cell embryos that successfully reached four-cell and morula stages did not differ significantly between the groups (Figure 1E). However, a transition from morula to blastocyst was accompanied by a higher embryo mortality rate in the KLH group (8.42% ± 1.64%) than in the control group (3.31% ± 0.94%; χ^2^ = 6.97, *p* = 0.008). As a result, the fertilization by spermatozoa from the KLH-treated males led to fewer embryos developing to the blastocyst stage compared to the fertilization by spermatozoa from the control males (Figure 1E). In addition, blastocysts in the KLH group had fewer cells than in the control group (Figure 2). From this, we concluded that the immune priming of males reduces their fertilization capacity and impacts the survivability and cell division of blastocysts.

### 2.3. Interaction between Spermatozoa and Seminal Fluid of The Immune-Primed Males Determined Embryo Implantation Efficiency

The antigenic challenge of males was expected to confer a signal to both the spermatozoa and seminal fluid. Seminal fluid presents antigens from the male to the female and, thus, could modulate embryo implantation and early developmental stages [13,14]. To address the effects of the seminal fluid from the KLH-treated males, we mated recipient females with vasectomized control or KLH-treated males as the donors of seminal fluid 2.5 days before embryo transfer. Approximately 17–19 two-cell embryos obtained by IVF with spermatozoa from the control (C) and immunized (Im) males were transferred to surrogate mothers exposed to the seminal fluid of the control (C) and immunized (Im) males. This resulted in four experimental groups: C–C (spermatozoa and seminal fluid from the control males; *n* = 14), C–Im (spermatozoa from the control, seminal fluid from the KLH-treated males; *n* = 12), Im–C (opposite to C–Im; *n* = 14), and Im–Im (spermatozoa and seminal fluid from the KLH-treated males; *n* = 7). Such an experimental design permits the identification of immune signals on spermatozoa and seminal fluid and their impact on implantation, gestation, and embryo growth.

**Figure 3:**
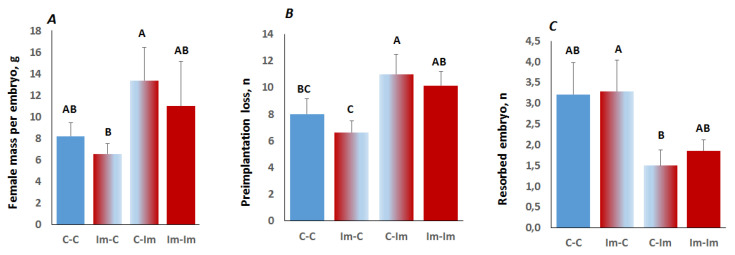
Female mice mass per embryo (mass/litter size) (**A**) and embryo losses at pre- (**B**) and post-implantation (resorption) (**C**) stages of pregnancy in the C–C, Im–C, C–Im, and Im–Im experimental groups. Note that the letters above indicate significantly different mean values (LSD test, *p* < 0.05).

There were no statistically significant differences in the body mass of pregnant females, the number of implanted embryos, or litter sizes between groups (Table 2). However, we noted significant between-group variations in female mass per embryo and the pre- and post-implantation embryo mortality. Notably, spermatozoa and seminal fluid effects on these traits were the exact opposite of one another in the C–Im and Im–C groups (Figure 3). Furthermore, pre- and post-implantation losses were oppositely affected in these experimental groups. In the Im–C group, the pre-implantation losses were lower than in the C–Im group (Figure 3B), while embryo resorptions were higher in the Im–C group than the C–Im group (Figure 3C). Considering all groups, two-way ANOVA revealed a significant effect of seminal fluid (mating with the control or KLH-treated vasectomized male mice) on all of these traits (Table 3). The effect of spermatozoa from control and KLH-treated male mice on these traits was insignificant.

Surrogate mothers mated with the KLH-treated vasectomized male mice had a larger mass per embryo compared to the control (12.48 ± 2.43, *n* = 19 vs. 7.38 ± 0.78, *n* = 28, respectively; *p* = 0.025, Student’s *t*-test). Mating with the KLH-treated vasectomized males also increased pre-implantation embryo losses: 10.57 ± 0.97, *n* = 19 vs. 7.32 ± 0.77, *n* = 28 (*p* = 0.007, Student’s *t*-test) for the females mated with the KLH-treated and control vasectomized male mice, respectively. However, females mated with the KLH-treated vasectomized males had significantly lower resorption rates than the females mated with the control vasectomized males (1.67 ± 0.55, *n* = 19 vs. 3.25 ± 0.44, *n* = 28, respectively; *p* = 0.02, Student’s *t*-test). Thus, we concluded that there exists cross-talk between immune signals conferred to the spermatozoa and seminal fluid, which guides pre- and post-implantation of IVF embryos.

### 2.4. Progesterone, Testosterone, and GM-CSF in Pregnant Female Mice

Pregnant females’ concentration of progesterone and testosterone in blood plasma and amniotic fluid (AF) did not differ significantly in the experimental groups (Table 4). Typically, for pregnancy, plasma progesterone and testosterone levels increased from the 5th to 16th day of gestation. Progesterone concentrations were 36.66 ± 3.82 and 55.27 ± 3.14 ng/mL on day 5 and 16, respectively (*p* < 0.001, Student’s *t*-test); testosterone levels were 0.07 ± 0.01 and 0.23 ± 0.02 ng/mL on day 5 and 16, respectively (*p* < 0.001, Student’s *t*-test).

Concentrations of both steroid hormones in amniotic fluid did not correlate with their concentrations in blood plasma on day 16 of gestation. The concentration of amniotic progesterone (11.49 ± 0.91 ng/mL) secreted by the fetoplacental complex was lower than plasma progesterone (*p* < 0.001, Student’s *t*-test). In contrast, amniotic testosterone (2.61 ± 0.09 ng/mL) produced by male fetuses was higher than plasma testosterone (*p* < 0.001, Student’s *t*-test).

Among studied humoral traits, only GM-CSF revealed statistically significant differences between groups of pregnant females (Table 4). The maximum level was detected in the group Im–C and the minimum level in the group Im–Im. The insufficient volume of amniotic fluid precluded the analysis of other humoral traits such as estrogens.

### 2.5. The Impact of Cross-Talk between Spermatozoa and Seminal Fluid of Immune Primed CD1 Male Mice on Embryo Development

**Figure 4:**
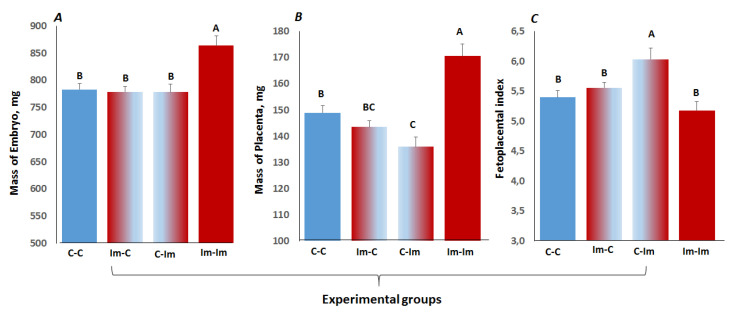
Embryo mass (**A**), mass of placenta (**B**), and fetoplacental index (**C**) on day 16 of pregnancy in surrogate mothers of the studied experimental groups. The letters above indicate significantly different mean values (LSD test, *p* < 0.05).

Finally, we contemplated how the immune signals conferred to the spermatozoa and seminal fluid of antigen-stimulated males affected embryo growth. First, we noted a significant increase in the masses of embryos and placentas on the 16th day of gestation in the Im–Im group compared to others (Figure 4A,B). The fetoplacental index was the highest in the C–Im group (Figure 4C) due to the fact that this group had the lowest mass of placenta compared to the other groups (Figure 4B). A significant gain in the embryo mass in the Im–Im group suggested an overall positive impact of the immune priming of males on IVF embryo growth, despite the lower IVF and blastocyst survival rates for these males (Figure 1D,E) and lower embryotrophic cytokine GM-CSF concentration in the Im–Im group (Table 4). This, in turn, further substantiates the importance of the cross-talk between the spermatozoa and seminal fluid of antigen-stimulated males on embryo growth.

**Figure 5:**
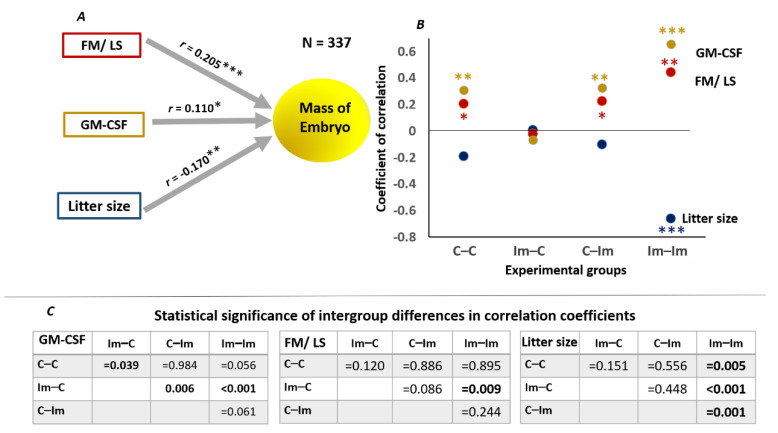
Correlation of embryo mass with maternal resource traits. (**A**) Coefficients of correlation for the whole data set (*n* = 337) of per-embryo female mass (female mass/litter size—FM/LS), litter size, and amniotic fluid GM-CSF (pg/mL) with embryo mass on day 16 of pregnancy. (**B**) Coefficients of correlation for the different experimental groups of FM/LS (red points), litter size (blue points), and amniotic fluid GM-CSF (yellow points). Significance of correlation coefficients in (**A**,**B**): * *p* < 0.05; ** *p* < 0.01; *** *p* < 0.001. (**C**) Statistical assessments of intergroup differences between coefficients of correlation (*p*-values were calculated with Student’s *t*-test).

Over the whole data set, embryo mass correlated moderately but statistically significantly with FM/LS (female mass/litter size), amniotic GM-CSF, and litter size (Figure 5A). However, we noted substantial differences in coefficients of correlation for individual groups that cannot be explained by the reduction in the data set (Figure 5B). For example, the correlation coefficients of embryo mass with the FM/LS, GM-CSF, and litter size were zeroed in the Im–C group. In the C–C, C–Im, and Im–Im groups, statistically significant correlations between the mass of embryos and the amniotic concentration of GM-CSF and the FM/LS ratio remained, while only in the Im–Im group was a canonical negative correlation between the number and mass of the embryos observed (Figure 5B). Pair-wise comparisons of the correlation coefficients suggest stronger associations between embryo mass and the FM/LS, GM-CSF, and litter size in the Im–Im group (Figure 5C). Thus, in addition to the gains in embryo mass, the immune priming of males plays a positive role in coordinating the mother’s resource allocation with regard to embryo growth and an optimal response to the growth-promoting cytokine GM-CSF. However, this requires that immune signals are conferred to both the spermatozoa and seminal fluid.

## 3. Discussion

The present study was carried out at the peak of the serum anti-KLH IgG immune response in antigen-treated male mice [8], which ensures exposure of maturing spermatozoa to a range of cytokines [18]. An interaction of pro-inflammatory cytokines with maturing spermatozoa reduces their number [19,20,21] and slows their transfer to the cauda epididymis [11]. This explains the observed negative correlation between spermatozoa concentration and serum anti-KLH IgG concentration. The observed decrease in spermatozoa concentration in mice on day seven after the injection of KLH was combined with their low fertility in vitro. The survivorship and cell divisions of blastocysts obtained by IVF from the sperm of KLH-treated male mice were also reduced, indicating an epigenetic signal conferred to spermatozoa by immunization.

Immune factors could modulate the epigenetic landscape of spermatozoa. First, the immune response involves inflammation, which triggers ROS formation [22,23]. Infections and ROS associate with aberrant DNA methylation causing male infertility [24], which could also explain the lower fertility of spermatozoa from immunized males. To that, oxidative stress in both male and female gametes could have a lasting effect on their offspring [25,26]. Interestingly, in humans, male subfertility associates with a loss of sperm DNA methylation in immune- and inflammation-related genes [27]. Second, based on the stages of spermatogenesis in mice, it could be suggested that the effects of antigenic stimulation observed on day seven after immunization might be due to the influence of immune factors on the process of sperm chromatin condensation [28,29]. Indeed, infectious or inflammatory processes adversely affect the histone to protamine reassembly and cause DNA damage to the paternal genome [30]. An improper histone-to-protamine ratio impacts spermatozoa fertility. It also affects the formation of blastocysts by assisted reproductive technology [31]. Increased mortality of the blastocysts could also explain the previously observed increase in the difference between the number of ovulated oocytes and live embryos in female mice mated with immunized male mice [5,6,32].

In addition to epigenetic modulation of spermatozoa, immune stimulation may also act on the seminal fluid. Thus, environmental cues may be transmitted from fathers to their progeny by either spermatozoa or seminal fluid. We transferred two-cell IVF embryos obtained using sperm from the control or KLH-treated male mice to surrogate mothers mated with vasectomized control or KLH-treated males to discriminate between these two possibilities. Transferring the two-cell IVF embryos from the control or KLH-treated male mice presents a paternal signal carried by spermatozoa to the female. Mating surrogate mothers with vasectomized control or KLH-treated males exposes them to a paternal cue from the seminal fluid.

The seminal fluid from the antigen-treated male mice significantly affected embryo implantation. This increased pre-implantation mortality but decreased post-implantation losses. The influence of immune signals present in seminal fluid on the uterus and its preparation for embryo implantation has been shown in several studies [13,14,33,34,35,36]. In the seminal fluid, these immune signals could cause an increase in the number of leukocytes, appearance of antigen-specific immunoglobulins, changes in prostaglandins, cytokines levels, etc. [8,37].

Seminal fluid is a significant regulator of the maternal humoral environment due to the modulating effect on the expression of growth factors and cytokines that determine embryo development and survival [35]. Factors present in seminal fluid induce the expression of embryotrophic cytokines (CSF1, GM-CSF, CSF3, IL-6, LIF, and VEGF) in the oviduct and uterus that have a positive effect on embryonic growth and development [33,38,39,40,41]. Mating females with males with excised seminal vesicles that lack seminal plasma impairs zygotic divisions and the development of blastocysts due to the activation of apoptosis [33]. 

Embryo growth was modulated by the paternal immune signals conferred to both the spermatozoa and seminal fluid. On the 16th day of pregnancy, embryo mass increased only in the offspring of the antigen-treated donors of spermatozoa and surrogate mothers exposed to the seminal fluid of immune-primed males. This agrees with a previous study showing that females mated with antigen-stimulated males in vivo produced larger fetuses than a control group [7]. Embryonic growth is highly dependent on the optimal allocation of resources between the growing fetus and the mother’s body. This is reflected in the correlation between the embryo mass and the litter size, maternal weight per embryo, and the concentration of embryotrophic factors, particularly GM-CSF. The highest values of the correlation coefficients were in the Im–Im group. Thus, we concluded that immune signals conferred to progeny through the seminal fluid and spermatozoa result in better coordination and more optimal allocation of the mother’s resources towards the embryo’s growth. Interestingly, knockout of the pro-inflammatory cytokine TNF-alpha flips the canonical litter size–embryo mass trade-off [42], substantiating the role of immune signals in the coordination of embryo development and growth.

The use of the IVF model allows for the evaluation of the differential effects of immune signals passed to spermatozoa and seminal fluid on the parameters of embryonic development. We showed that the antigenic challenge of male mice reduced IVF rates and the survival of blastocysts. In conjunction with the exposure of surrogate mothers to the seminal fluid of immune-primed males, it led to a gain in embryo growth due to the more optimized resource allocation on the part of the mother. Although these results did not reveal the paternal immune signal per se, they point to possible scenarios for the realization of the effects of antigenic challenge on the reproductive function of men, which includes viral infections. For example, COVID-19 interferes with spermatogenesis, reduces sperm quality in moderately infected patients, and poses health risks to their offspring [43,44,45]. Thus, the IVF model can be effective for studying the effects of antigens of different natures on male reproduction and the development of their offspring.

## 4. Materials and Methods

### 4.1. Animals and Immunization

All studies were performed at the Centre for Genetic Resources of Laboratory Animals, Institute of Cytology and Genetics of the Siberian Branch of the Russian Academy of Sciences. We used specific pathogen-free (SPF) male and female CD1 mice, 12–14 weeks of age, to account for genetic diversity [46]. An artificial photoperiod (14L:10D), stable temperature (22–24 °C), and humidity (40–50%) were maintained. Animals were housed in same-sex groups (5 per cage) in individually ventilated cages (OptiMice, Animal Care Systems, Centennial, CO, USA). All animals had ad libitum access to water and granulated mouse food (SNIFF, Germany). Experiments were performed under protocols approved by the Bioethics Review Committee and the Animal Care and Use Committee of the Federal Research Centre Institute of Cytology and Genetics. All animal handling and experimental manipulations followed the Federal Ministry of Health (2010/708n/RF), NRC, and FELASA recommendations.

CD1 male mice (12–14 weeks old) caged individually were intraperitoneally (i.p.) immunized (*n* = 48) with KLH (Sigma, SRP6195). The dose was 50 μg of KLH per 100 μL of saline. Control males (*n* = 48) were injected with 100 μL saline. Seven days later, these male mice were used as sperm donors or were vasectomized and mated with surrogate females. Oocytes for fertilization by control and immunized male mice sperm were obtained from 20 and 22 females, respectively. A total of 26 and 19 females mated with vasectomized control and immunized male mice, respectively, were used for embryo transfer.

### 4.2. Spermatozoa Parameters: In Vitro Fertilization

For the analysis of spermatozoa, the right cauda of the epididymis was transferred into a tube containing 500 μL of Hanks solution (Sigma) prewarmed to 37 °C without Mg^2+^ and Ca^2+^. Five to six sectional cuts were made, and the samples were incubated for 20 min at 37 °C. Spermatozoa parameters: concentration (number/mL), size (μm^2^), and motility (% of total spermatozoa moving at a path velocity of ≥30 μm/s and a progressive velocity of ≥15 μm/s) were measured using the automatic Mouse-Traxx sperm analyzer (Hamilton Thorne, USA). Five fields (20 μm deep Micro Cell Counting chamber) were examined at 4× magnification for each sample.

In vitro fertilization was performed with spermatozoa from the control (*n* = 18) and KLH-treated males (*n* = 18). CD1 male mice were administered KLH or saline. Seven days later, spermatozoa were collected from the cauda epididymidis for IVF. For this, cauda epididymidis were placed into a 100 µL drop of human tubal fluid (HTF, Cosmo Bio, Japan), covered with mineral oil (Sigma–Aldrich, St. Louis, MO, USA), and incubated for one hour at 37 °C in 5% CO_2_.

Virgin CD1 female mice were superovulated by i.p. injection with 5 IU of pregnant mare’s serum gonadotropin (Folligon, MSD Animal Health, Boxmeer, The Netherlands ). Then, 48 h later, they were i.p. injected with 5 IU of human chorionic gonadotropin (hCG; Horulon, MSD Animal Health, Boxmeer, The Netherlands). Cumulus oocyte complexes collected from the oviduct ampulla (15–17 h post-hCG injection) were placed into 200 µL of fertilization drop containing HTF. Spermatozoa (3–5 µL from the pre-equilibrated HTF drop) were added to the fertilization drop and incubated for 3–4 h. Fertilized oocytes were washed through four drops of the HTF medium and further cultured in 80 µL of HTF covered with mineral oil (Sigma) at 37 °C and 5% CO_2_ to the two-cell (E1.5) stage. The fertilization rate was calculated as the ratio of two-cell embryos to the total number of oocytes. Two-cell embryos were cultured to the blastocyst stage in the KSOM medium (Cosmo Bio, Tokyo, Japan) at 37 °C and 5% CO_2_.

The number of embryos reaching the stage of four cells, morula, and blastocysts was counted daily. Embryo viability was assessed by the proportion of embryos that reached the blastocyst stage. For evaluation of the cell number, the blastocysts were washed in a 60 μL drop of phosphate-buffered saline (PBS), fixed in 4% formaldehyde (15 min), and stained with 1 µg/mL of Hoechst-33258 in PBS for 10 min. Stained blastocysts were analyzed using the Axio Observer D1 (Carl Zeiss, Jena, Germany) inverted phase-contrast fluorescence microscope.

### 4.3. Experimental Groups, Embryo Transfer, Embryonic Development

Embryo transfer (ET) was performed by flank laparotomy in pseudo-pregnant CD1 female mice recipients (12–14 weeks old), which were obtained by mating with the control or KLH-treated vasectomized CD1 males. Recipients were anesthetized by isoflurane (Aerran, Baxter Healthcare Corp., USA) using the Matrix VIP 3000 vaporizer (MID MARK). In vitro fertilized embryos (17–19 per recipient) were transferred into the left uterine horn. After the transfer, exposed tracts were placed back into the abdominal cavity, the peritoneum was sutured, and the skin was closed with wound clips. Recipients were then kept alone in individually ventilated cages (OptiMice, Animal Care Systems, Centennial, CO, USA) to recover from anesthesia. On day 16, the pregnant females were weighed and decapitated. Samples of blood plasma were collected and stored at −80 °C until use. The amniotic fluid of each embryo from the same uterus was pooled and stored at −80 °C until use. The embryos were then separated from the placentas and weighed. The fetoplacental weight ratio (a ratio of the embryo to placenta weight) was used as a proxy for placental efficiency [47].

We formed four experimental groups to differentiate the effects of spermatozoa and seminal fluid from immunized males on embryo growth and development. Between 17 and 19 two-cell embryos from the control (C) or KLH-treated (Im) males were transferred to the recipient females mated 2.5 days prior with vasectomized control (C) or KLH-treated (Im) male seminal fluid donors. Thus, the four groups were as follows: C–C (control spermatozoa, control seminal fluid; number of females—14), C–Im (control spermatozoa, immunized male seminal fluid; *n* = 12), Im–C (immunized male spermatozoa, control seminal fluid; *n* = 14), and the Im–Im group (*n* = 7).

### 4.4. Testosterone, Progesterone, and GM-CSF Immunoassays

The insufficient volume of amniotic fluid did not allow for the determination of the concentration of estrogens. Thus, we limited the analysis to progesterone, testosterone, and GM-CSF. The sensitivity of the testosterone and progesterone assays were 0.087 ng/mL and 0.15 ng/mL^−1^, respectively. The intra- and inter-assay coefficients of variations (CV) were 8.2% and 5.6%, respectively, for the testosterone assays and 7.6% and 4.3%, respectively, for the progesterone assays. The GM-CSF concentrations in amniotic fluid were determined with the GM-CSF Mouse ELISA assay (Abcam, Inc., Cambridge, UK). The minimum detectable dose of GM-CSF was less than 9 ng/mL^−1^. The intra- and inter-assay CVs were 5.2% and 3.9%, respectively.

### 4.5. Statistical Analysis

The Student’s *t*-test was employed to compare the means of normally distributed traits. A two-way ANOVA test was used to elucidate the statistical significance of the effects of immunization of sperm donors or seminal fluid donors (vasectomized males). The χ^2^-test assessed the differences between the fertilization rate and embryo mortality. Correlations between embryo mass and maternal indexes and between males’ anti-KLH IgG and sperm concentration were evaluated by Pearson correlation coefficients. Data are presented as the mean ± SEM.

## Figures and Tables

**Table 1:** Spermatozoa characteristics of the control and immunized (KLH) CD1 male mice.

Traits	Control (*n* = 9) Mean ± SEM	KLH (*n* = 9) Mean ± SEM
Size	32.8 ± 1.6	33.3 ± 1.4
Elongation	63.1 ± 1.1	63.6 ±0.8
VAP	94.3 ± 7.0	93.5 ± 3.9
VSL	66.2 ± 5.3	66.8 ± 2.8
VCL	190.0 ± 11.4	184.7 ± 7.5
ALH	14.8 ± 0.5	14.6 ± 0.3
BSF	39.5 ± 0.3	38.9 ± 0.33
STR	64.7 ± 1.6	64.9 ± 0.5
LIN	34.5 ± 1.4	35.1 ± 0.6

VAP—average path velocity, VSL—straight-line velocity, VCL—curvilinear velocity, ALH—amplitude of lateral head displacement, BSF—beat cross frequency, STR—straightness (VSL/VAP), and LIN—linearity (VSL/VCL).

**Table 2:** Body mass of pregnant CD1 female mice, number of implanted embryos, and litter size on day 16 of pregnancy.

Groups	*n*	Female Mass Mean ± SEM	Implanted Embryos Mean ± SEM	Litter Size Mean ± SEM
C–C	14	46.4 ± 1.5	10.5 ± 1.3	7.23 ± 1.09
Im–C	14	48.7 ± 1.6	11.9 ± 1.2	8.57 ± 0.76
C–Im	12	46.1 ± 2.3	8.1 ± 1.6	7.18 ± 1.46
Im–Im	7	44.2 ± 1.8	8.2 ± 1.1	7.17 ± 0.91

**Table 3:** Two-way ANOVA of the effects of male immunization (injection with KLH) on the per-embryo female mass and pre- and post-implantation embryo losses.

Factors	Female Mass/Litter Size		Pre-Implantation Losses		Post-Implantation Losses (Resorption)	
	F1,44	*p*	F1,44	*p*	F1,44	*p*
Spermatozoa donor (KLH vs. control)	0.76	0.39	0.95	0.33	0.07	0.79
Vasectomized male (KLH vs. control)	4.74	0.03	7.01	0.01	5.42	0.02

**Table 4:** Progesterone (ng/mL), testosterone (ng/mL), and GM-CSF (pg/mL) in blood plasma and amniotic fluid (AF) of surrogate mothers on days 5 and 16 of pregnancy.

Traits	Day	Groups			
		C–C Mean ± SEM (*n*)	Im–C Mean ± SEM (*n*)	C–ImC Mean ± SEM (*n*)	Im–Im Mean ± SEM (*n*)
Progesterone	5	37.7 ± 9.4(14)	36.8 ± 6.5(14)	31.8 ± 6.5(12)	43.6 ± 2.8(6)
Progesterone	16	50.9 ± 5.4(14)	57.7 ± 7.1(14)	56.5 ± 6.9(12)	56.8 ± 3.0(7)
Progesterone (AF)	16	10.3 ± 2.0(14)	11.0 ± 1.7(14)	13.8 ± 1.9(9)	11.9 ± 0.5(7)
Testosterone	5	0.08 ± 0.02(14)	0.08 ± 0.02(14)	0.06 ± 0.01(12)	0.05 ± 0.01(7)
Testosterone	16	0.22 ± 0.05(14)	0.28 ± 0.04(14)	0.19 ± 0.01(11)	0.20 ± 0.05(7)
Testosterone (AF)	16	2.57 ± 0.16(14)	2.74 ± 0.22(14)	2.44 ± 0.15(12)	2.72 ± 0.04(7)
GM-CSF (AF)	16	96.1 ± 16.0^AB^ (12)	127.5 ± 15.9^A^ (12)	110.5 ± 26.6^AB^ (9)	57.3 ± 7.9^B^ (6)

Different superscript letters indicate significantly different mean values (LSD test, *p* < 0.05).

## Data Availability

The data that support the findings of this study are available from the corresponding author upon reasonable request.
